# Particle Swarm Optimization with Scale-Free Interactions

**DOI:** 10.1371/journal.pone.0097822

**Published:** 2014-05-23

**Authors:** Chen Liu, Wen-Bo Du, Wen-Xu Wang

**Affiliations:** 1 School of Electronic and Information Engineering, Beihang University, Beijing, People's Republic of China; 2 School of Management, Beijing Normal University, Beijing, People's Republic of China; University of Maribor, Slovenia

## Abstract

The particle swarm optimization (PSO) algorithm, in which individuals collaborate with their interacted neighbors like bird flocking to search for the optima, has been successfully applied in a wide range of fields pertaining to searching and convergence. Here we employ the scale-free network to represent the inter-individual interactions in the population, named SF-PSO. In contrast to the traditional PSO with fully-connected topology or regular topology, the scale-free topology used in SF-PSO incorporates the diversity of individuals in searching and information dissemination ability, leading to a quite different optimization process. Systematic results with respect to several standard test functions demonstrate that SF-PSO gives rise to a better balance between the convergence speed and the optimum quality, accounting for its much better performance than that of the traditional PSO algorithms. We further explore the dynamical searching process microscopically, finding that the cooperation of hub nodes and non-hub nodes play a crucial role in optimizing the convergence process. Our work may have implications in computational intelligence and complex networks.

## Introduction

Biological entities and natural processes have always been the source of inspiration for the resolution of many real-world applications, one of which is to search the optimum of a problem under some certain conditions via the evolution of a population, namely the nature-inspired optimization algorithm. The advantages of self-adaption, self-organizing and self-learning in natural-inspired optimization approaches allows us to address many challenging problems pertaining to complex systems that cannot be solved by traditional methods. Hence much effort has been dedicated to the nature-inspired optimization algorithms in the last decades, as summarized in several reviews for succinctly recent advances on this topic [1]–[3].

Particle swarm optimization (PSO) is a typical nature-inspired optimization algorithm proposed by Kennedy and Eberhart [4], which is inspired by the social behavior of swarms such as bird flocking or fish schooling [5]. In nature, a bird usually adjusts its movement to find a better position in the flocking according to its own experience and the experience of birds nearby. In PSO, each artificial particle has its own position and updates its velocity according to the attractiveness of its best previous position and the best previous position of its direct interacted particles. Consequently, a group of interacted particles (the population size is commonly selected in the range 20–50 [1], [6]–[9]) cooperate with each other to search for an optimum in the solution space.

Since PSO was proposed, it continuously draws attention in the field of nature-inspired optimization. Shi and Eberhart [6] introduced the inertia weight coefficient to damp the velocity of particles iteratively and thus the scope of the search can be better controlled. Several years later, Clerc and Kennedy [7] proposed a strategy for the placement of constriction coefficients and derived a reasonable set of parameters. They proved that PSO with constrictions is algebraically equivalent to PSO with inertia weight coefficient from a theoretical way. The “constriction” version PSO has been the canonical particle swarm algorithm today. Moreover, Mendes and Kennedy [8] indicated that a particle is not only simply influenced by the best neighbor so that they introduced the fully informed PSO, in which particles are affected by all their neighbors. Kennedy et al. [9], [10] found that PSO with large neighborhood would perform better on simple problems, while PSO with small neighborhood might perform better on complex problems. Peram et al. [11] took distance into account and proposed the fitness-distance-ratio based PSO that combats the problem of premature convergence, in which particles move towards nearby neighbors with better fitness instead of the global best solution. Due to its effectiveness and simplicity in implementation, PSO has been widely applied in solving many practical optimization problems such as transportation [12], biomedicine [13], [14], power systems [15], [16], communication system [17], [18], electronics and electromagnetics [19], [20].

However, in previous literatures about PSO, the particles are completely [4], [21] or regularly interacted [8], [10]. Since the discovery of small-world phenomenon by Watts and Strogatz [22] and scale-free property by Barabási and Albert [23] a decade ago, it has been realized that most real networks are neither fully connected networks nor homogeneous regular networks, but of small-world and scale-free topological characteristics. Much evidence has demonstrated that the structural properties play key roles in dynamical processes taking place on complex networks [24]–[26], e.g., in the evolutionary game dynamics, cooperators cannot survive on fully-connected networks, but some cooperators may have the chance to establish clusters to against the invasion of defectors and survive on a regular lattice. Strikingly, scale-free topology can lead to the emergence and domination of cooperation [27]–[29]. Such previous findings prompt us to wonder how scale-free topology that captures the interaction pattern among particles affects the PSO and if scale-free topology can offer better performance of the optimization process. To answer these interesting questions, we proposed a SF-PSO, in which scale-free network that incorporates the diversity of individuals is exploited to better mimic the real situation and improve the traditional PSO. We find that the SF-PSO balances the convergence speed and the solution quality, resulting in a much higher optimization performance than traditional PSO. We substantiate our findings and validate the SF-PSO by systematic analysis associated with a variety of benchmark functions used in the literature.

## Materials and Methods

### Canonical PSO Algorithm

We adopt the framework of the canonical PSO and give a brief introduction. Suppose the size of the population is *N*, where each particle 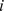 has a position 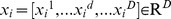 and a velocity 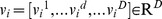 in the *D*-dimensional solution space. Here 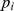 is particle 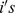 best previous position and 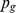 is the best previous position of the best performer in 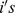 neighborhood. 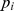 and 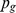 is updated iteratively. The particles are manipulated according to the following equation: 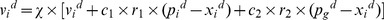
(1)

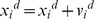
(2)where 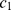 and 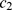 are the acceleration coefficients, 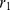 and 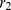 are two uniformly distributed random numbers which are separately generated in the range [0,1]. 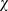 is the constriction coefficient to control the convergence speed of the population [6].

The first component of Eq. (1) stands for the previous velocity that provides particle the momentum to search the solution space. The second component is the “self-cognitive” part that particle learns from the best previous position of itself. The third component, known as “social-cognitive” part, represents how particle optimizes the position by learning from the best previous position of its neighbors. Following common practices, 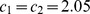 and 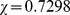 [1], [8], [30], [31].

### Benchmark Functions

Benchmark functions are often used to evaluate the performance of optimization algorithms [8], [10]. The formula, the number of dimensions, the admissible range of the variable, the optimum and the goal values are summarized in Table 1. We use all these representative functions to test the optimization performance of our algorithm in comparison with traditional PSOs.

**Table 1:** Optimization benchmark functions.

Function name	Formula	*D*	Range [*x_min_, x_max_*]	Optimum	Goal for function
Sphere	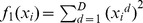	30	[−100,100]^D^	0	0.01
Rosenbrock	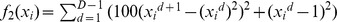	30	[−30,30]^D^	0	100
Rastrigin	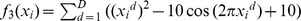	30	[−5.12,5.12]^D^	0	100
Griewank	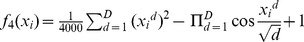	30	[−600,600]^D^	0	0.05
Quartic	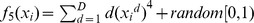	30	[−1.28,1.28]^D^	0	0.05

All functions are implemented in 30 dimensions and are minimization problems. The optimum is the best solution for the function. The goal for function is used to evaluate the optimization is successful or not. The algorithm is run for 5,000 iterations. If the goal is not met by that time, it is considered that the goal will never be met and the optimization is unsuccessful. 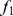 and 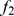 are unimodal functions; 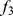 and 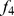 are multimodal functions; 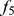 is the noise test function and is also unimodal function.

### Comparative Study of Different Topologies

**Figure 1:**
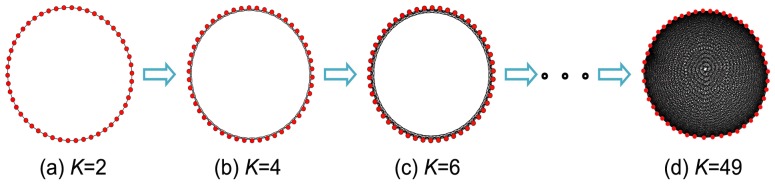
Networks with different values of *K* (the average degree). The network density grows from left to right. Here (a) is a ring-like network and (d) is the fully connected network.

**Figure 2:**
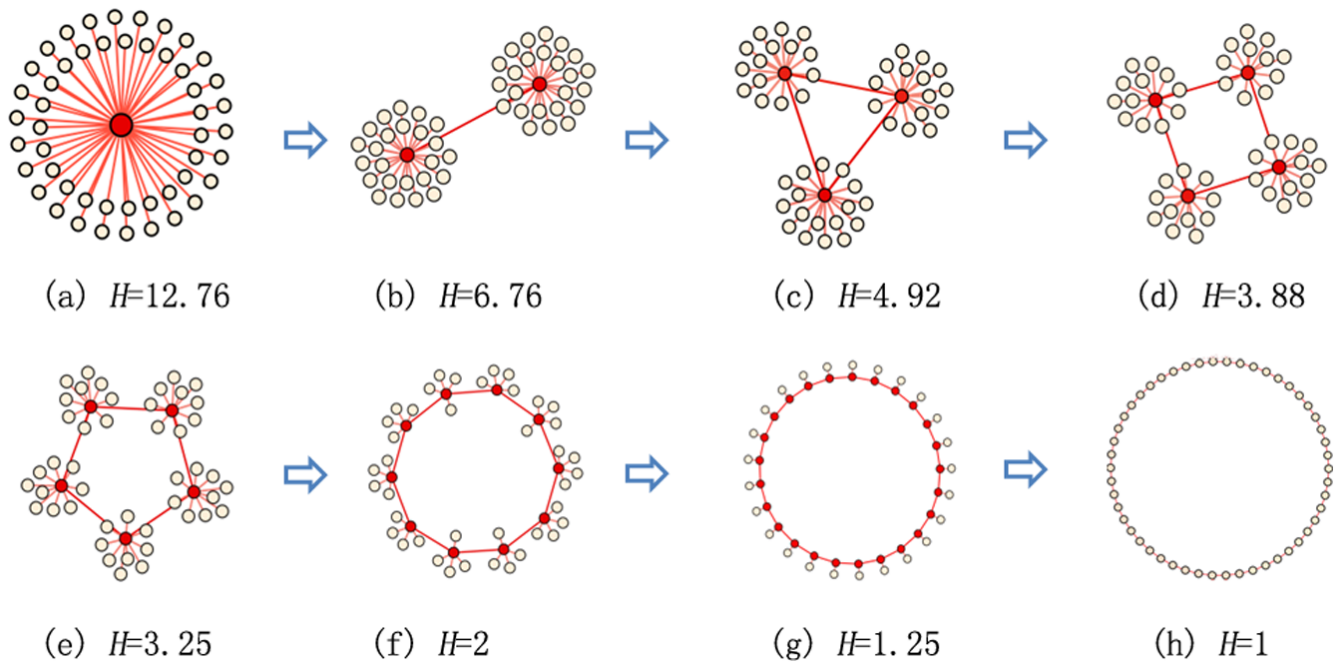
Networks with different values of *H* (the network heterogeneity). 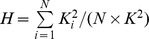
, where *K* is the average degree of the topology and 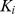 is the degree of particle 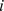. The network heterogeneity decreases from left to right, from up to bottom.

In previously established PSOs, the population is assumed to be completely or regularly connected. However, the commonly observed structural characteristics in complex networks motivate us to explore the effects of various topologies on the performance of PSO. Firstly, we use the topologies from ring to fully connected to test the performance variation of regular topologies with different network densities (Figure 1). Secondly, we design a graphical scheme that enables a continuously transformation from a heterogeneous star-like network to a homogeneous ring-like network to examine the effect of network heterogeneity (Figure 2).

All the topologies are tested in the framework of PSO for the optimization of five benchmark functions (Table 1). We use the comprehensive performance criterion S in Refs. [8], [10] to evaluate the performance of different topologies. Since different functions are scaled differently, it is impossible to combine raw results from different functions. In this situation, we standardize the results of each function to a mean of 0.0 and standard deviation of 1.0. Thus, all results from different functions are normalized to the same scale. As all of these functions involve minimization, a negative result after standardization is better than the average. After standardizing each function separately, we can combine them and find the average as the value of S.

**Figure 3:**
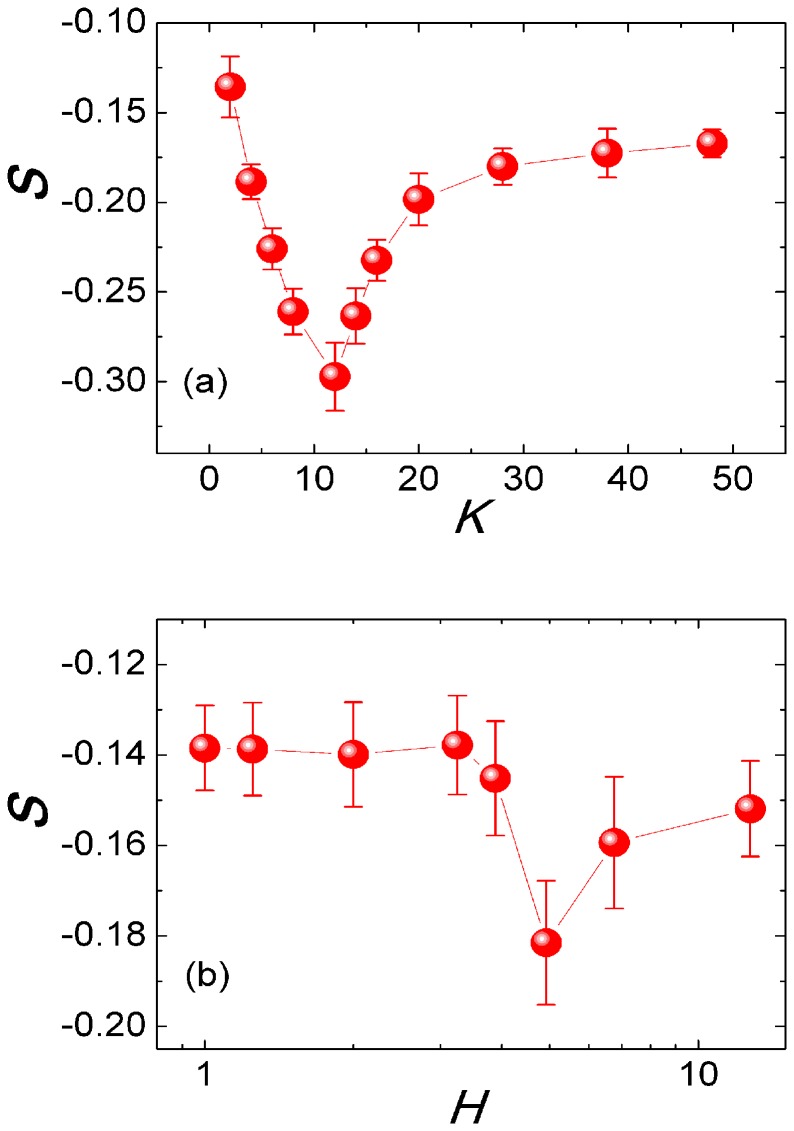
The comprehensive performance criterion *S vs.* the average degree *K*, (a). The comprehensive performance criterion *S vs.* the heterogeneity *H*, (b). The size of the population *N* is 50 and each point is averaged over 100 times. Algorithm runs for 5,000 iterations each time.

As shown in Figure 3(a), neither the fully connected topology nor ring topology performs the best. The information dissemination of fully connected topology is too fast to be able to escape from the local optima. The ring topology, on the contrary, prohibits the achievement of convergence, accounting for its bad performance. Topology with medium density can achieve the best result by making a balance of convergence speed and optimum quality (e.g., *K* = 12). From Figure 3(b), we can also find that a topology with a moderate heterogeneity performs better (e.g., *H* = 4.92). In high-heterogeneous topology, e.g. star-like network, a hub particle holds the dominate position and it can influence most of the population due to its large degree. Particles will soon converge to the hub, resulting in the tragedy of premature convergence. In homogeneous topology, e.g. ring network, however, no particle has a significant effect on the whole population. The convergence speed is too slow to induce good performance. Based on these preliminary results, we infer that network with moderate density and heterogeneity facilitates PSO.

### SF-PSO Model

In 1999, Barabási and Albert found the scale-free property in real networked systems and proposed the well-known Barabási–Albert (BA) scale-free model whose generating mechanism can be described as “growth” and “preferential attachment” [23]: starting with *m_0_* connected nodes, at each step a new node is added with *m* (*m<m_0_*) edges that link to *m* different existing nodes and the probability 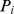 that a new node connects to node 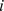 is related to 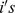 degree 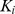:
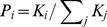
(3)where *j* runs over all the existing nodes. An important characteristic of scale-free networks is the degree distribution 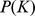, which is defined as the proportion of nodes in the network with degree *K*, and it can be described by a power law with a simple form: 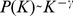. A variety of empirical researches have revealed that numerous real networks are actually scale-free networks [32], [33] and scale-free properties have a significant impact on network dynamics [24]–[26].

**Figure 4:**
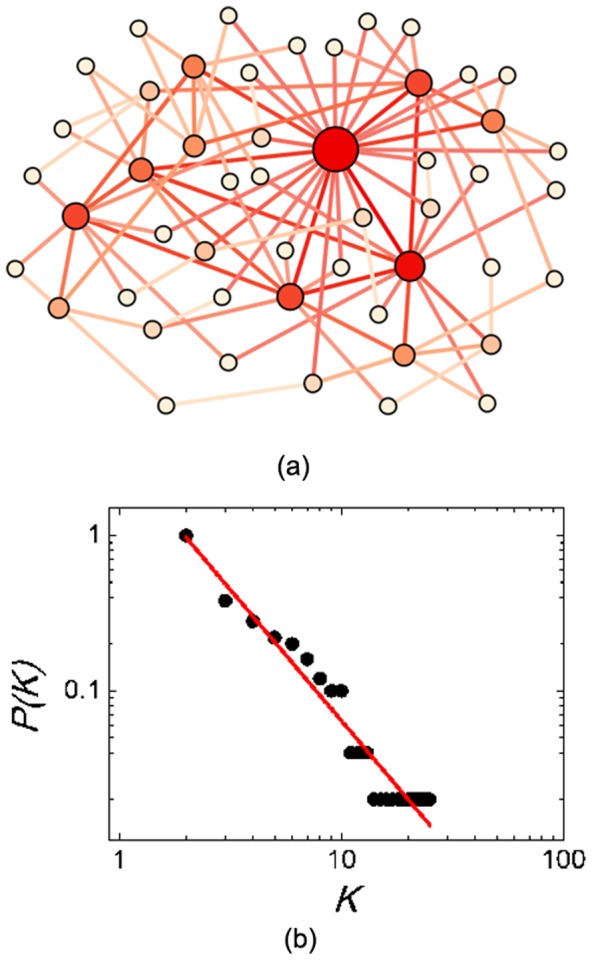
A typical BA scale-free network with: *m_0_* = 4, *m* = 2 and *N* = 50, (a). The cumulative degree distribution of the BA scale-free network, (b).

A typical BA scale-free network is shown in Figure 4(a) and its cumulative degree distribution is presented in Figure 4(b). We can find that scale-free networks are neither dense as fully connected networks nor sparse as ring networks. Meanwhile, scale-free network is heterogeneous but not as heterogeneous as star-like network. In the following, we adopt the scale-free network to represent the individual interactions in PSO, namely SF-PSO, and further investigate its performance.

## Experiments and Discussions of SF-PSO

### Optimization Performance

The SF-PSO is compared to PSO with fully-connected network (F-PSO) and PSO with ring network (R-PSO). The results are displayed in Table 2. We employ the main solution quality criterion *R*, which is the best solution at the end of the optimization, to examine the performance of algorithms. F-PSO obtains the best solution quality on 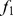, while SF-PSO ranks only second to it. SF-PSO obtains the best solutions on 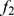, 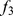 and 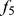, which means that SF-PSO can achieve high quality solutions on unimodal function (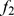), multimodal function (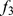) and noise function (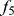). R-PSO achieves the best on 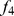 while SF-PSO is just a little behind. Here we also examine the comprehensive performance criterion *S*. The result turns out that SF-PSO is remarkably better than other two PSOs, where SF-PSO is 25% smaller than F-PSO and 54% smaller than R-PSO. Obviously, SF-PSO's performance is outstanding according to solution quality criterion *R* and comprehensive performance criterion *S.*

**Table 2:** Performance of three PSOs.

		F-PSO	SF-PSO	R-PSO
*R*	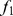	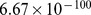	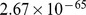	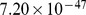
	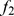	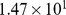	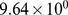	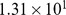
	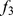	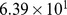	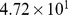	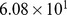
	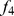	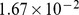	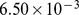	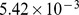
	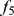	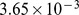	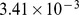	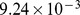
*S*		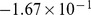	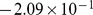	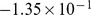

The size of the population *N* is 50 and each value is averaged by 100 times. Algorithm runs for 5,000 iterations each time. The results of SF-PSO correspond to the average over 10 network configurations with *m_0_* = 4, *m* = 2.

According to the “no free lunch” theorem [34], any elevated performance over one aspect of problems is offset by performance over another. Since SF-PSO performs better in solution quality and comprehensive performance, there must be a cost in some other aspects. Next, we examine another important performance criterion of optimization algorithms, the convergence speed *Q*, which is the number of iterations required to accomplish the goal in Table 1. The results of three PSOs are shown in Table 3. It is found that F-PSO is of the fastest convergence speed while R-PSO is of the lowest. Due to the moderate network density and network heterogeneity, SF-PSO converges a little slower than F-PSO but faster than R-PSO. It is the “fate” of SF-PSO according to “no free lunch” theorem.

**Table 3:** Convergence speed of three PSOs.

		F-PSO	SF-PSO	R-PSO
*Q*	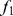	**313**	502	695
	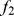	**595**	665	861
	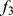	**157**	266	410
	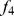	**282**	457	656
	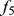	**357**	475	855

The size of the population *N* is 50 and each value is averaged by 100 times. Algorithm runs for 5,000 iterations each time. The results of SF-PSO correspond to the average over 10 network configurations with *m_0_* = 4, *m* = 2.

**Figure 5:**
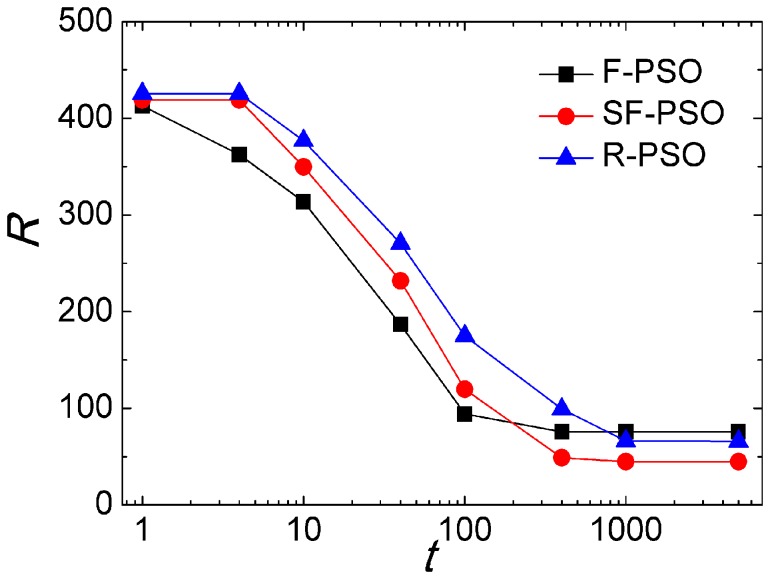
Optimization process of three PSOs on Rastrigin function. The size of the population *N* is 50. The network parameters of SF-PSO are *m_0_* = 4, *m* = 2. Algorithm runs for 5,000 iterations. We have also examined other benchmark functions, and the results are alike.

Figure 5 shows the optimization process of three PSOs on Rastrigin in detail. We see that, F-PSO converges faster than the other two PSOs in the beginning. However, the population converges on a poor local optimum due to its “overspeed”. For the R-PSO, the convergence speed at the early stage is the slowest, but the final solution quality is better than F-PSO. SF-PSO balances the convergence speed and solution quality, yielding the best performance in the comparison.

Next we investigate the optimization microscopically to uncover the underlying mechanism in SF-PSO that facilitates the optimization process.

### Search Process of SF-PSO

**Figure 6:**
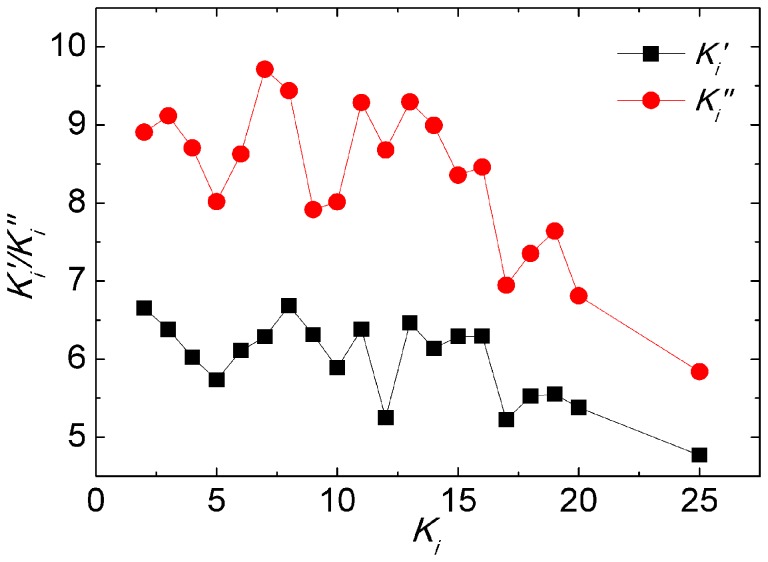
The black square symbols stand for the average degree of particle 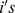 neighbors 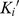 and the red circle symbols stand for the average degree of neighbors that particles learn from 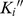. Each data is averaged by 100 times on 10 network configurations with *m_0_* = 4, *m* = 2 and *N* = 50. Algorithm runs for 5,000 iterations each time.

We suspect that the high-degree hubs and low-degree particles may play different roles in the optimization process. To confirm our speculation, we firstly explore what kind of neighbors that particles tend to learn from. We define 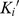 as the average degree of particle 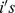 neighbors and define 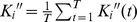 as the average degree of neighbors that particle 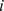 learn from during the whole evolution, where *T* is the maximum iteration of the optimization and 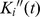 is the degree of neighbor that particle 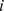 learn from at iteration *t*. Figure 6 shows that 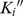 is significantly larger than 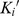, indicating that particles are more likely to learn from the neighbors with larger degree in their neighborhood, in other words, particles tend to learn from hubs. In the following, we use the scale-free network in Figure 4 to represent the interaction topology of particles.

**Figure 7:**
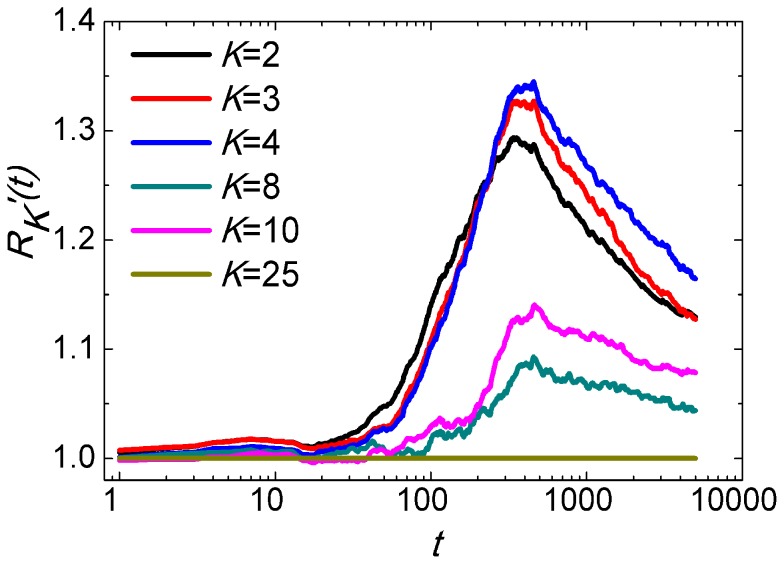
The variation of relative solution quality 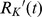. Each data is averaged by 1,000 times. The network parameters of SF-PSO are *N* = 50, *m_0_* = 4, *m* = 2. Algorithm runs for 5,000 iterations each time.

Since particles tend to learn from hubs, it is natural to examine whether the hubs have high solution quality or not. We define 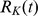 as the solution quality of particles with degree *K* at iteration *t*. Figure 7 represents the relative solution quality of particles with degree *K*: 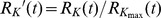 iteratively, and *K_max_* is the largest degree of population. We can find that at the early stage of the optimization, there is insignificant solution quality difference among particles with different degrees. As the optimization process evolves, the gap emerges. Since the largest degree of the population is 25, the relative solution quality of the particle with degree 25 is always 1. The relative solution qualities of particles with medium degrees (*K* = 8, 10) are between 1.0 and 1.1 at the end of optimization, while the solution qualities of particles with small degrees (*K* = 2, 3, 4) are above 1.1. It can be interpreted that hubs can obtain much more information and explore larger space due to its large degree.

**Figure 8:**
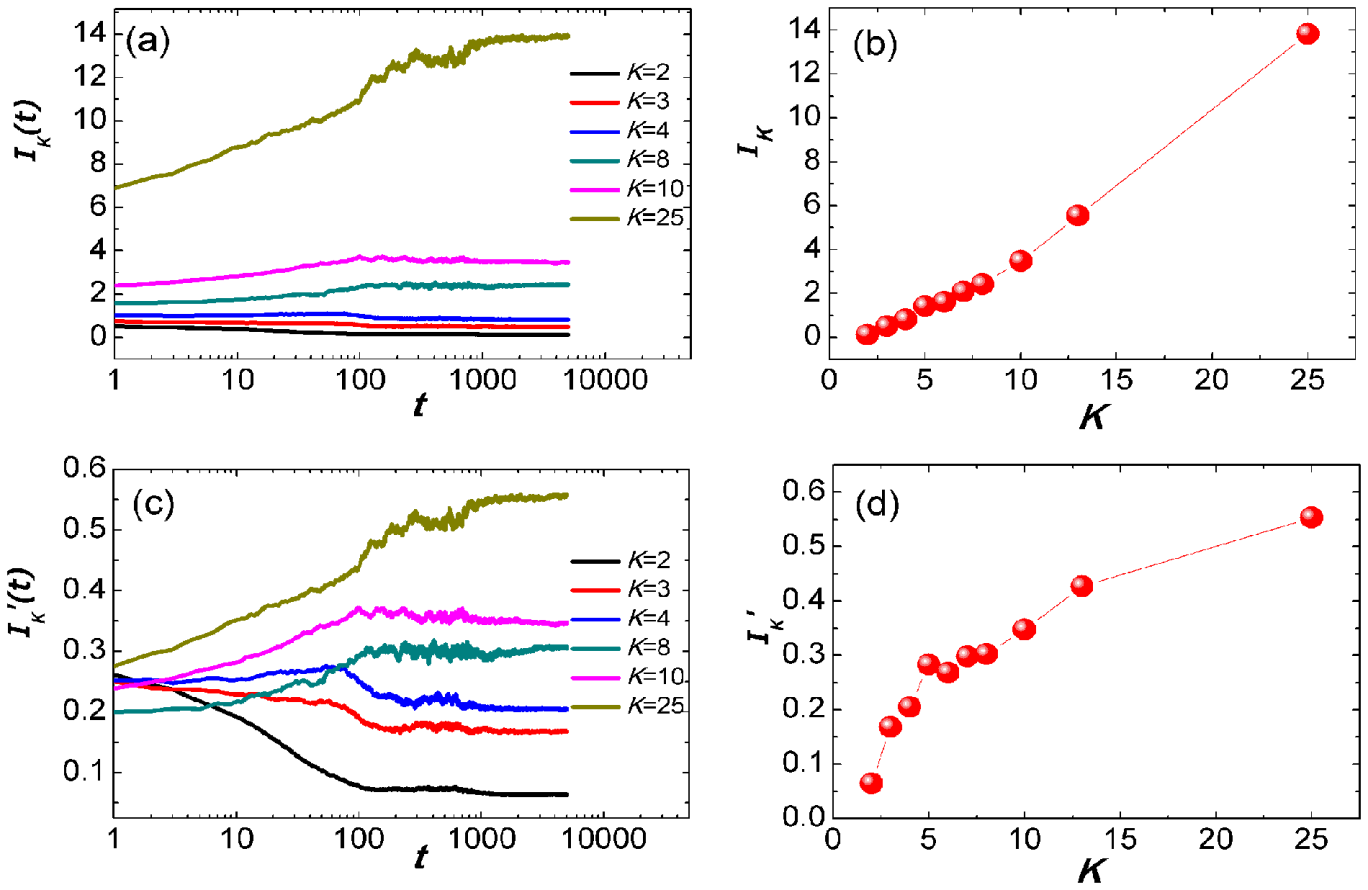
The information dissemination ability of particles with different degrees. (a) 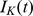 *vs.* iteration *t*. 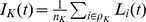, where 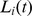 is the times that particle 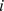 is learned by its neighbors at iteration *t*, 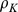 is the collection of particles with degree *K* and 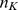 is the size of 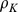. (b) 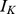 *vs.* particle's degree *K*. 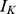 is the average performance of particles in the steady state. (c) 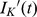 *vs.* iteration *t*. 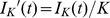. (d) 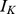 *vs.* particle's degree *K*. 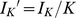. Each data is averaged by 1,000 times. The network parameters of SF-PSO are *N* = 50, *m_0_* = 4, *m* = 2. Algorithm runs for 5,000 iterations each time.

Further, we focus on the influence of particles with different degrees on information dissemination. The information dissemination ability of particles with degree *K* at iteration *t* is defined as 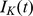. Then, 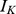 denotes the performance of particles' information dissemination ability with degree *K* in steady state. Figure 8(a) shows the variation of information dissemination ability iteratively. It is found that, the advantage of hubs become increasingly significant during the evolution. Figure 8(b) shows that particles' performance on information dissemination in the steady state. Apparently, the performance of hubs is better than that of low-degree particles. Thus, it is natural to claim that hub particles play a crucial role on the information dissemination of PSO. To get rid of the influence of the degree factor, we further define two averaged indices: 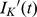 and 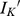. As Figure 8(c) shows, in the beginning of the evolution, 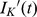 is around 0.25 for all particles. However, with the increment of time step, 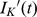 of medium-degree particles is still around 0.25 while high-degree particles and low-degree particles are obviously divergent. From Figure 8(d), we can also find that high-degree particles outperform low-degree particles in steady state.

As we know, the optimization process of PSO (as well as many other nature-inspired optimization algorithms, such as GA [35], ACO [36] and SA [37]) is actually the process of finding “better solutions”. If the particles find more “better solutions”, the population is more active and facilitates the optimization of PSO. In this regard, we investigate the particles' ability on finding “better solutions”, in other words, particles' contribution to maintaining the population's activity.

**Figure 9:**
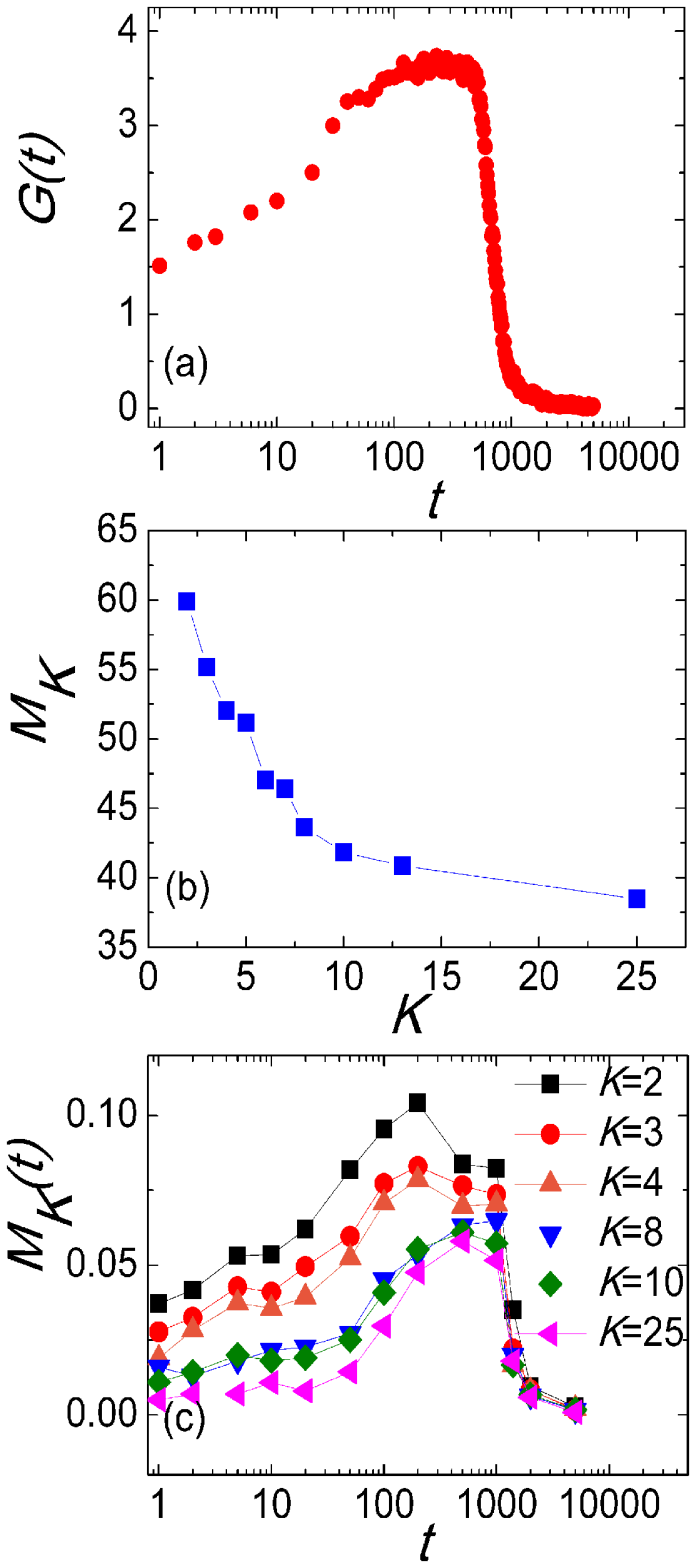
Particles' contribution to population's activity. (a) 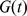 *vs.* iteration *t*. 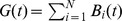, where 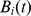 is particle 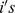 ability of find a better solution at iteration *t*. The value of 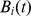 is 1 when particle 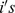 solution quality at iteration t 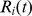 is better than the previous best solution found by its neighbors 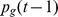. On the contrary, the value of 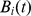 is 0. (b) 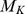 *vs.* particle's degree *K*. 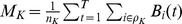, where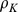 is the collection of particles with degree *K* and 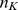 is the size of 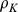. (c) 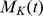 *vs.* iteration *t*. 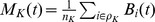. Each data is averaged by 1,000 times. The network parameters of SF-PSO are *N* = 50, *m_0_* = 4, *m* = 2. Algorithm runs for 5,000 iterations each time.

Here, we define 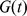 as the population's contribution to its activity at iteration *t*. As Figure 9(a) shows, 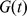 firstly rises and then declines. At the beginning of the optimization, all particles are initialized with random positions and random velocities. As the optimization process evolves, particles will gather to the better regions in solution space according to the rule of PSO. If it is easier for particles to find better solution, 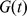 will rises. When the optimization close to the optimum, it is hard to find better solutions and the value of 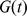 declines.

To distinguish the contribution of particles with different degrees, we define 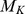 as the average contribution of particles with degree *K*. Surprisingly, we find that the value of 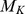 monotonously decreases with the increment of degree, indicating that low-degree particles make more contribution to maintaining the activity of the population. The only chance for a particle to find a better solution is to defeat all neighbors according to the definition of 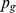 in Eq. (1). Although a high-degree hub particle can collect more information through more links to obtain a considerable good solution, it is harder to find a “better solution” since it has too many rivals. As to a low-degree particle, it is just the opposite. Figure 9(c) shows the evolution of 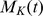. The variation tendencies for all particles are well accordance with 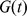 in Figure 9(a). Besides, the contribution of particles monotonously decreases with the increment of degree. It is just the perfect combination of guiding hub particles and low-degree particles who keep the activity of population that makes outstanding performance of our SF-PSO.

### Conclusion

We have incorporated scale-free topology into the particle swarm optimization, attempting to improve the optimization process with respect to its solution quality and convergence velocity. We have found that the scale-free topology that captures the diversity of individuals leads to the balance between the solution quality and the convergence efficiency, which outperforms the traditional particle swarm optimization algorithm based on either fully-connected graph or regular graph. Interestingly, the much better performance of our approach is attributed to the cooperation between hub nodes and non-hub nodes, where the former is of strong ability to ensure high solution quality and guides the evolution direction, while the latter helps to maintain the activity of the population for exploring the solution space and escaping from local optima. Our findings suggest the paramount importance of exploiting the diversity in population for achieving better evolution pattern of swarm, which has many implications in computational intelligence and controlling a variety of dynamical processes taking place on complex networks.
